# Supplementary material for: MaNrtB, a Putative Nitrate Transporter, Contributes to Stress Tolerance and Virulence in the Entomopathogenic Fungus Metarhizium acridum
Source: J Fungi (Basel). 2025 Feb 1;11(2):111. doi: 10.3390/jof11020111 (PMC11855974; doi:10.3390/jof11020111)
Supplement: Supplementary file 1 [file jof-11-00111-s001.zip › jof-3352404-supplementary.pdf]

MaNrtB, a nitrate transporter, contributes to stress tolerances and virulence in the entomopathogenic fungus *Metarhzium acridum*

Jia Wang[1,2,3,4], Yuneng Zou[1,2,3,4], Yuxian Xia[1,2,3,4*], Kai Jin[1,2,3,4*]

1 Genetic Engineering Research Center, School of Life Sciences, Chongqing University, Chongqing 401331, PR China; 202326021033@cqu.edu.cn (J.W.)

2 Chongqing Engineering Research Center for Fungal Insecticide, Chongqing 401331, PR China

3 Key Laboratory of Gene Function and Regulation Technologies Under Chongqing Municipal Education Commission, Chongqing 401331, PR China

4 National Engineering Research Center of Microbial Pesticides, Chongqing 401331, PR Chi-na

* Correspondence: yuxianxia@cqu.edu.cn (Y.X.) and jinkai@cqu.edu.cn (K.J.)

The genomic DNA sequence of the high affinity nitrate transporter NrtB MAC_03189 [Metarhizium acridum CQMa 102]

AAAGTTCCTGTAC**GAAGAAAAGTTGATGCACAC**AGCACCATCGCGAATAAGGTCTGTAGGAACCTTGAATGATTCCACAGGAACACCTCCAATGAC
AACGTCGCTAAGAGGCAGGCAGTCCTTCAATCCCCAGCCCTCCTTGTCCTCGACCTGGTGTCGCGGGCTCTTGATACCCTGACCACGGGTGAATAACT
GAACTCCGGTAATATCAACAGAGTATACAGTAGCACCATCATTGGCTAGCAAAGCAGCGAGAGGTCGGCCGTTTACTTCGGAGCGGTTAATGACGGTG
ATAGTTTTGCCAAACAGGCGATTCCCGGCTGCCAAAATGGGGTTGTAAATCTGAAGATATTCCAAGATCTTAACAACAGCCAGTGGAGTGCATGGCAA
AATAGACTTCTTCCGGTTCTCCGGGGGATCCAGGAATCGGATATTGTGATACATGTTGTAAAGATACTTGTGGCGCATACCCTCGACATCTTTGGCAAG
CTCTACAGTCTCCTGGACGTACTTGTCATGTGTGGGGTTCTGGGGGAAGATAGGATAGTAAACGATGATGCCGTCAACAACGTCATCTTCATTGGCTTT
CGTGATCTCCTCCTCGAGAAGATCTTTTTCAACAGTGCGAAGGTCAAAGTTGAAACCACTGTTGAAGGACGTCAGCGTCTACAGGTCATCACCTTGG
GATGCAAAACATATCCTGCTCTACTCACTTCTCCTCGCAAGTCTTTTTTGACCACTGGGCGTACTGAACCGCAGCGGGATCACCGTTTGCCAAGAAGG
CAACAAGCGTCGGTGCTTTGGGGTTGCCTCCCTGGACTTTGGCCAGCGTCTCTTTGACTTCGGTCAAGAGATTCTTAGCAATGGTCTCTGCAGTAATG
ACCTTGCAGGTCTTGGGAACTTCTGGCGAAGCCATTATCTGAGCACCAAGTCCGTATTTGCGTCGAATTGTTTACCATCCAGCTGGGCAGACAACAGT
CGGATGAGGAGTAAGTCTTGATTAAGAACCCCGCCAATGTAGCCTTGAACAAGTCGAAGCTCTACATCGTCACTGGTCAGTGAATAAGGCGGATTGG
GTCATGGGGGCAGTAGACGAACAATTCAGGTTGTGATTCCCTGCACGTCCTATAATCAACAGGCCAGGTCAACAATGCTCTCACTCGTGAAGCTTCAA
CTGTCTATTGGAGATGCATGATTACGTCGAATCGCTGAGGAAAAAAAAATATGCCCCTCCAAAACCTTTGATGCCGCAGTTGCCCCTCTTACTAACCAT
GGACTAGCGCCAGTAAGCAAGTAGTAAATGGTGGAGACGAGCCAATGCACCTTCCCTTGCTTGAACCAACTTTTGACGTTCGGCCTTGAGCCTCAAC
AACGTTGCATCTAGACTCGCTGGCGGGTGCACGACTTATAAATCCTTCCTTTTCACCAATTTTGAAGAAATCGGTCTTGTTCGTTTGTCTATTAGAACC
ACGTGTTGGGGATTGAACAATTCCGCATGATGCCCATCATTGTTCGTGGAAAACCAGAGTCTGGAGTCATCACCAATGGTGAGAAAAAAGGCCCAAA
TCCGCAGAAAATCCGCAG**TCGAGCAGCTTCAACTTATA**AAATTTTACAGC**CGATGTCATAGCCTTATGGT**TTTTGAGAAGCTTC**AACGCCACCAA**
GAAGGTATTCTTCATTTCGACAATATGTGGTGTCGCGCGCTGCTCTTGCGACCGATTGATGTGCGCGGAGTTCATCGGTGGCCAAGCTCTCCGTTCAG
ATCGCGGAACAGAATCCATGTGATGCAAGTCATACTTGTGGACTGAGTCCTTCATGTGATTCTCATCTTCAGTTGGACGATCGAGGTTTACGTCAAGCT
CAACACTATACACCGGATACCTCCTCTGGCGGGCCAAGTTCTGCATCCCGAGTTCGTCCTGACAGCAATCAACAGAACAATGCCATTGCCAACACGTC
ACGCGCAGAGTCGGTCTCTCTGATATATACTCATAGAGTAACATTTTATCTCGCATACCCCGGGTTTTTATCTCATCTGCCGAAGCGTTCTTCTCCGCGG
GTGTACAAGTTGATAAGCTTCCCCAGGTCAGTCGGTTATCTTTGCAGTGTGGAGTCGCACTGGCGCCGGTGTCCAACTCGTCCAGATAAGGTCAGACC
AACCAACATCACCGCTTTGAACACCCCTTTTCTGATAGGATAATTGTCCAATAGAATTCATTGGATCGGATATTCGCTCGAATCCGTCGATAACATGCCT
GCAATGGCAAGTTCGATTAACATTTCAAACACCTTTGAGGGAGATAATTTTCATCACGTGAACCTTCCAAGCTACAGGAGACATTACCCGCCCCCTTT
GAGGGTTACTTGGTCAATAAATAGGACCACGACTATGCGGTATTGTAAGACGTGTCTTTCTGATGAATTGAGCGGTGAATCAACGACAAGAGACCGCA

AAAACGAAAAGAGATAGCATATGTAGGAGTATCAACAAAAATCTTTTTATTGTAAGTGTGTGATGCGGTTGGGATCTTGCCCGGGCTAGCTGGCTCATT
TCTTATCAGTGGCCGCGACAATTGTCAGAACCCAAGCGTATCAGCGCCGCCAGAGGTAGTTGGGTACGATGTCGATCTGGGCGTACTGTACGATGGGC
CTTGTTATCATATGTACCCTGAAAACGCCGGGTGTATCCGGCAATTGAATGAGACGGCCTTAACGCCCTAGTGTCGAAGGACTGGTCCATTCGCAGTC
CCAACAAATCGGGACCCAAGATCTTTGCTGGTTGGCTATCGAATCTAGATAAGCATTCAGCCCGGAGATGCCTGGAGATGCTTAAATCGGACCCTGGC
TGGATGTCTTTTTGAAGTTGATTATTATCGCAATGAACCAGAGTATTTAGAGGTCACGA**CGCCAACAACTCAAATCAAT**ATCGTATCGAATAGCGAAA
TTCCACTGTAAGGTGATTTCGAGCACTGCTTTTCAAAGCTATTTGACAACTTTCCAAAAAATGGGCTTCAATATATCTTTGTTATGGAAGACCCCGATG
GTCGACCCCATCAACAAGAAAGCTCGAAGTATACCTGTTCTGAACGTAGTTGATCCGTATGGACGAGTAT**TTTTCTTCTCTTGGATGGGG**TTCATGC
TTGGGTTTTGGGCATGGTACGATGACCTTTCTTTCCCTTACCTCTCTTTTTCCCTTCCTTCCAGGCTAATGTCGTCTGCACAGGTAC**ACGTTTCCTCCA**
TTGTTGACAGTCACGATTAAAAAGGACCTGCACCTCAGCGCCGCCGAGGTAGCCAACTCCAACATTGTATCCCTCTGTGCTACTCTACTTCTACGATT
CGTCGCCGGACCCCTCTGCGACCAGTTCGGCTCCCGGAGAGTCTATGCCTCACTCCTCCTCCTGGGCTGTCTACCGGTCGGCCTCGCGCCCCTCGTCA
AGACCGCCAACGGCCTCTACGTCTCCCGATTCTTCATTGGTATTCTCGGAGCCACGTTTGTCCCATGCCAAGTCTGGTGCACCGGCTTCTTCGACAAG
AACATTGTCGGCACGGCCAATGCTCTCTCTGGAGGATGGGGCAACGCCGGCGGCGGCATCACCTACTTCATCATGCCGGCCGTCTTCGACTCCCTGGT
TGCTTCCCAGGGCATGGCTCCGTCCAAGGCATGGCGAGTCACCTTTGTTGTCCCCCTCATTTGCCTGATCGCCTGTGCTCTCGGCATGCTATTCCTCTG
CCCGGACACCCCCCTGGGCAGCTGGGAAGAGAGGTCGCAAAAGCTCCAAGAAAACCTGGACCAATACAGCCCCACGAGCACCACGGCCGTCAACA
CGCCTCACATCCTCAGCGAGCCGCCGAGTCGAGACGTAGAAAAGGCGGAAGAATTCGACGAAGACTCGAAATTCTACAAGCAACCGTCTGCCATCT
CCTTGTCGGAAGCCGTGGCCATTGCCCAGGCGGAAACCGTGGTCAAGCCCAGCTTCAAGGACTCTCTACCCGTCATGCTCTCCCTACAGACCCTCTT
CCATGTCGCCACGTACTCGTGTTCATTCGGCGGCGAGCTGGCCGTCAACTCCATCCTCAGCTCCTACTACAAGGCCAACTTTCCACACTTGGACCAGA
CCAAAGCCAGCAACTACGCCGCCATCTTCGGCTTCCTCAACTTTGTCACACGGCCGCTCGGCGGCGTCGTGGCAGACATCCTGTACAGAATGTCCGG
ACAAAACCTGTGGACGAAAAAGGCCTGGATCACCATGGCCGGCCTCCTGAGCGGGGCGTTGCTCATCATCGTTGGAAAGGTTGATCCTTCGGAAGCC
AATGGCCGCGACATCGGCACAATGGTAGGCCTCGTCACCGTCGCCGCCTTCTTCATAGAGGCAGGCAACGGTGCCAATTTCGCCCTCGTGCCTCACGT
CTATCCTGCCGCCAACGGCGTCTTATCCGGGTGCACCGGCGGCGGCGGCAACCTGGGCGGCGTGGTATTCGCCATCATCTTCAGGTTCATCGATCACG
GCAGCGGCTACGCCACTGCCTGCTGGGTCATCGGCGTCA**TTCATATTGCCGTCAACTTG**GCAGTCTGTCGGATTCCGCCGCTGCCAA**AGGGCCAAG**
TCGGCGGGCAGTAAGCATACTCTCAGTCAGGGTCCCAAAATCGCATCATGTATATATTGGATTTGAAAGTTTTCACCTAGCGAGTAGCGAGTTTTCAT
TGGCCTGGGCCATTTCTTCCAAACAATGCCATTTTCTTTTTTTTTAATTCTTCTATTTTTTTTCCAGCGTTGCCTGCTTGCAGTGCTCTTGACTCGTCAAA
CGTCACTCGGTCTCAAGCATTCAGTCGCCTGACCAACCCCCCCAACGTGACGAAGGTCAGACACACTGTCCTCACGTGCTGAGACAAATAACCGAA
CAATGGTGAACTCTTCCCGCCTTGTCGCAGCCCTTGGCTTGTGAACTGCCTTGTGAAACACAGCCGATCCCGAGTGCCACCCCGCCAAAAGGCGTTT

GCAGATCCGATACAGAGCCAAGCCTTGATCGCCGAGATCTCTCCTTAACTGCGAATTGGCTTGCGGAGACTCAATCAGTTAGCGCGTTGTGAGGCAA
GAGGGAAACTCCACAAGCCATTTTGCAGTTAAAGAGATGTTTGTTTGGGCTCGGCCCTGTAACCAGCCGTGTGCAATCTGACATTGGACTTGACGCTT
AGAATGGGCTTCCGCCGGCCGTTTCTCCCGTGACTGTACTCCGTACGTACGTATGTGCGTATGTACTCGGGAAATTACACTTGCTTGTCAGAATATTGG
ATGAAACCCTAGCACGAGCCAAGAGCGGGTCTTCCTGACACCTGTTTGTCTCTGCATCACCCACAGACATGTCTTACATTCCAGCGCGGTGTAGCACT
AGTACTCCGTACTGATCATGAATAACATGGTCAGGCGAGGCCGATGGCAACTGGGTCAAGGTTGATTGAGGCTGAACGTATGTAGTCAAAGTCGCAC
GCTTTGCGTAACTACGGCAGGGCGTAGCTGACGAAATGGATAAGACAGTACACCGTAGATTCCATGTGGGATTCAGAGACACACAACACTAGAGGGG
CTCTTAAAGATGCACGCAGTGGTCAATTAATGTGATGAAATTGTGTGTACGTAGCACCTCGATCCGGGCTTAGCATTAACGGAAACGTTCATCAAGCCA
TCAACTAATGTAGCCAAACAGAATGGGTTCCGCGCATCACTAGCAAGAATGACCCGAAACT**TTCCAATCAGTGCCAATATC**AACCCATGTACCGGTG
CCCATTAGCAGAGCG**CACCATGTATCGTATCGTAT**ATGTGTAAGAGAAGCCCACGACCAGATTTAAAAAAAAAAAAACACGCGTAACCACCCACTG
TTACCGATTCTTTGCTTACAGAGCAAGAACGGCGGCAACAACGCCCAGAATGGTGAGTGAGCCGACGCGAACGGCGTTGGCAGCAGCAGTAGGGGT
AGCAATAGTACTGGGAGCAGTGGTAGTGGGCTTGAAAACAGCAGTGCTGGAAATGACAACGGGAGCAGACTGGCTGGATGAGGTCTTGAAAACCA
AAGTGGTGGTGGTGTTGGCAGGAAGGGTGGTGTTGTACTTGGTGGTCTTGGACTGAGTGCTGGTTGTGGCGGGAGCAGAAGTCGTCGCGACGGAAG
TGCTGGCGGCGGCAGTGCTAGTGACTGGAGTGCTGGTAGTAGACAAAGTCACAGTCTTGGTGCCGTAAGACGTGGTAATGGTGATGCTGCCGCTCGA
GCTGCCAGAGGGCTGGCTGCCAGATGCCTTGATATGGAAAGGGTTGGAGTACTGGAAGATGGTGTCGGGCTGGCTCTCGAGGCGGAAGACAAGACC
ATATACAGCCTTGTCACCGAGAGAAGCGCCGACCGTCCATGTGTAGGTCTTGGCGCTGTTCTTGACACCGGCTATAACATCGTGTTAGGAAACAGTCT
CGTCTTGGCCATGTCGATCATTGTCATTTTGCGCCAACTTACAAGCGATATCAGCAAGCTTGACCTGAGTATTCTGGGTAGCGCCACCGATCAGCTCAA
TTTTGACGGTACCATCAAGGTATTTGGCGGGAGCGTCCCAGGTGATAGTATAAGGAGAGCCAGCAACAATAGTCTCGCCAGACTTGGGGGTGTAGATG
GGGTCGAAGTCGGCGGCCTGGGCAAAGGCAGTGGCGGCAAAAGCCAGAACAGCGGCGACAGAGAAACGCATGTTGATGGGTCAACAAATACAAAC
GTATTATGTCAAATGTCGAGAGGTCAAATATTGACCGGAGCGAAAGAGCGACTGGGAGAGTGTTTTAAAGAGCGTGGGTGGCACAAAGATGCCTAGA
TAAAGGAGCGACAGTGGTTGAAGAAAGGAAGGTGCGAGTCGTGACCGAAACGAAGAGGTGAAGTGCAATAATAGTTTGGGGAGGCACTGGGGTAT
TCTCCGTCGCAGCACGTAGTCGACATCAAGCTTGTTGGCCGTGGACCCATAGGTTGACGGGGCGTGCCTTGCCAACAATGGCGCCCGACCGTTGGTT
CCTGGACCATTTCATCAGCGGGCTTGCTCGGTGGCTGCTGATTCGGGAGTGCCACCAGACATAGGCAGCAGGCACCACCCGCTGCACCACCCACCCA
CAGCACGTCAAGTCATGACGGTTCTTGGCGGGCCAAAAATGTCACTGGGGTGGAGCCCGTGTCAGGGGAAAAGCAAATTACACCCAAGAATGGGGG
CCTGCTATTGGCCAGTGCAGGGCCTGCGCGGGGCTGAGCATGCATCCTTTGACGGGTTGGTAGTGGCAGTCAAGGGTCGCGCGATTGACATCGTGGG

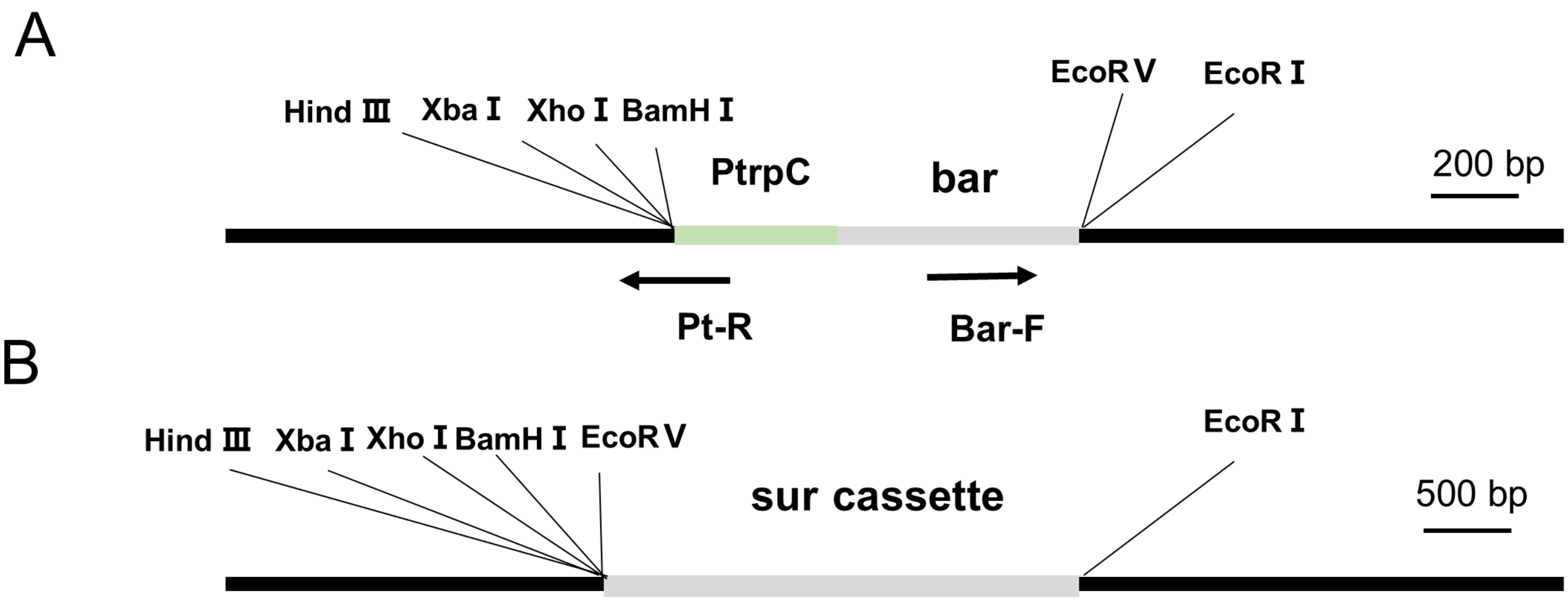

Figure S1 The schematic diagrams of pK2-PB and pK2-sur vectors. (A) *Hin*dIII/*Xba*I and *Eco*RV/*Eco*RI-restricted pK2-PB vector to insert the left border and right border of *MaNrtB*, respectively. Pt-R and Bar-F are universal primer located in the *bar* cassette. (B)The eGFP and TtrpC sequences were enzymatically ligated to the 5' end of the sur cassette, resulting in the formation of the PK2-eGFP-SUR vector. This construct can be used for the construction of any gene C-terminal fusion EGFP vector.

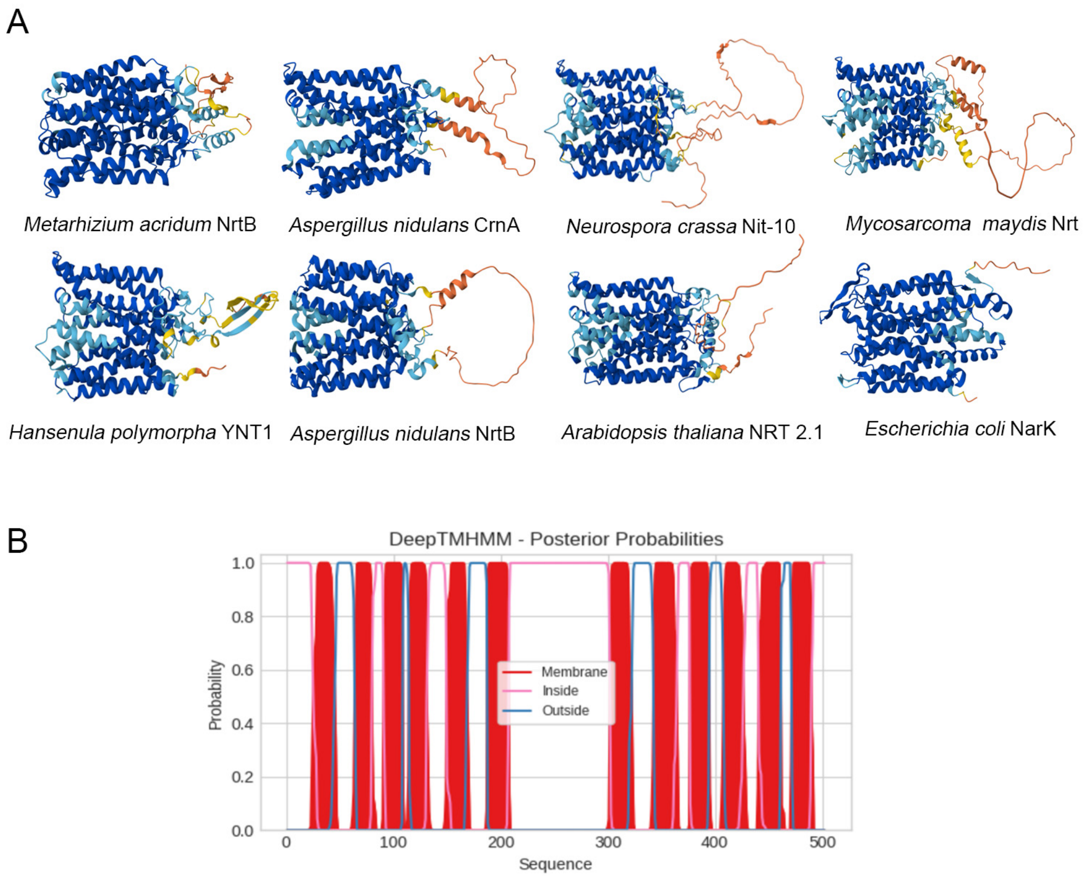

Figure S2 Structure prediction of MaNrtB protein. (A) Comparison of the three-dimensional structure of the nitrate transporter protein Nrt in different organisms. The three-dimensional structures of these Nrt homologous proteins are similar. Acesseion ID: *Aspergillus nidulans* CrnA (XP_658612.1), *A.nidulans* NrtB (AAL50818.1), *N.crassa* Nit-10 (XP_957430.2), *M.maydis* Nrt (XP_011390345.1), *H.polymorpha* (XP_018213600.1), *A.thaliana* NRT 2.1 (NP_172288.1), *Eschericha coli* (CAD6015891.1). (B) Transmembrane structure analysis of MaNrtB protein. The 12 trans-membrane domains were highlighted with red color.

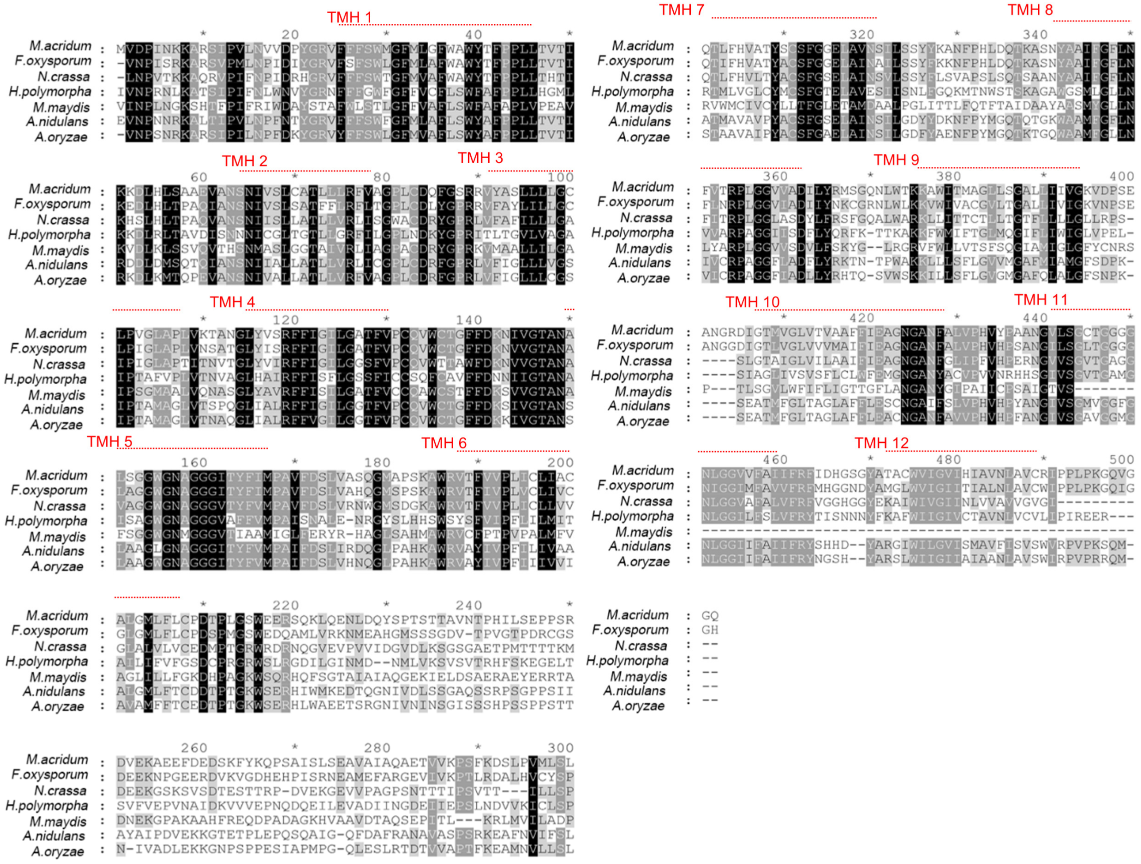

Figure S3 Alignments of amino acid sequences of the NrtB protein with reported homologues in other fungi. The MFS structural domain was marked by a red line. Transmembranal helices (TMHs) 1 to 12 are indicated above the sequences by red dotted lines. Accession numbers are as follows: *M. acridum* (XP_007809529.2), *N.crassa* (XP_957430.2), *F. oxysporum* (XP_018232784.1), *M. maydis* (XP_011390345.1), *A. oryzae* (EIT78908.1), *A. nidulans* (XP_658612.1).

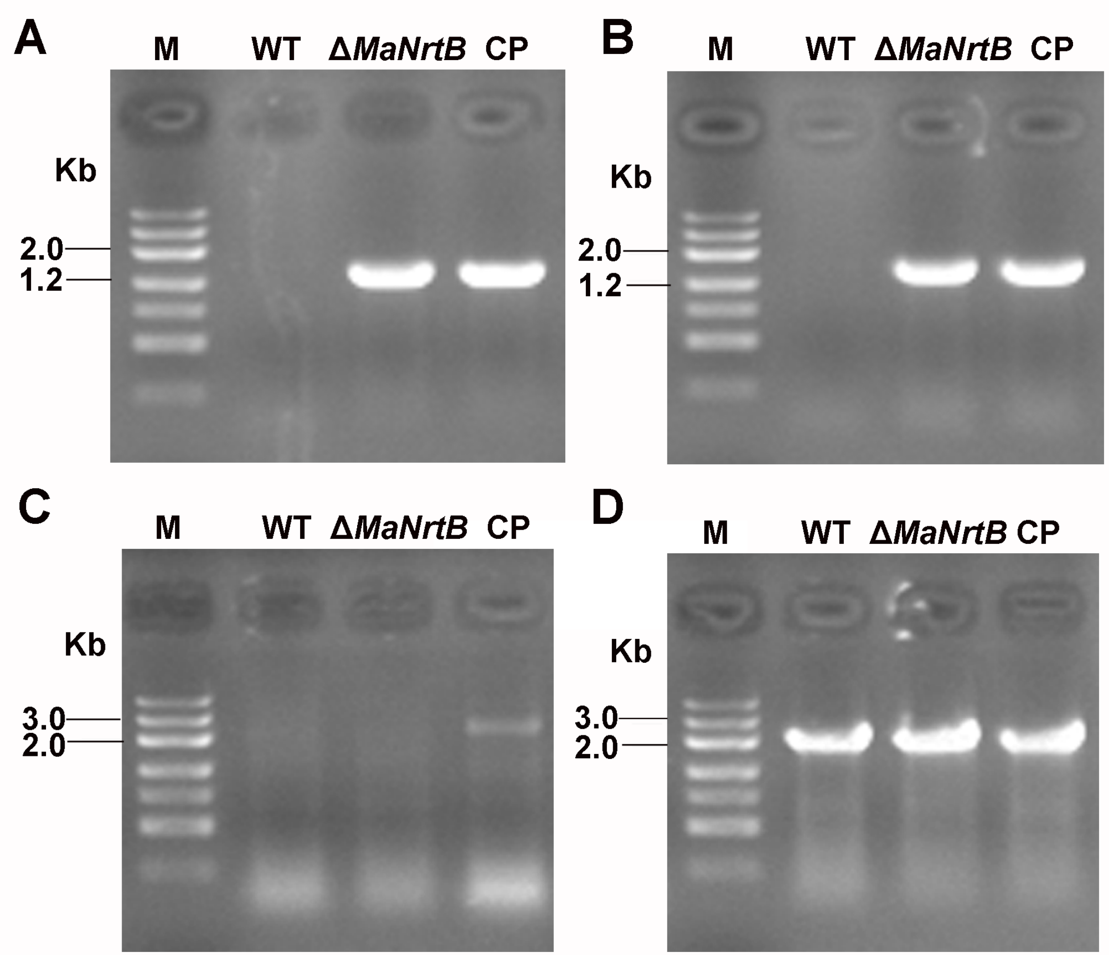

Figure S4 Validations of the Δ*MaNrtB*, and CP strains by PCR using primer pairs of NrtB-VF/Pt-R (A), Bar-F/NrtB-VR (B), NrtB-CP-VF/GFP-VR (C), and TEF-F/TEF-R (D).

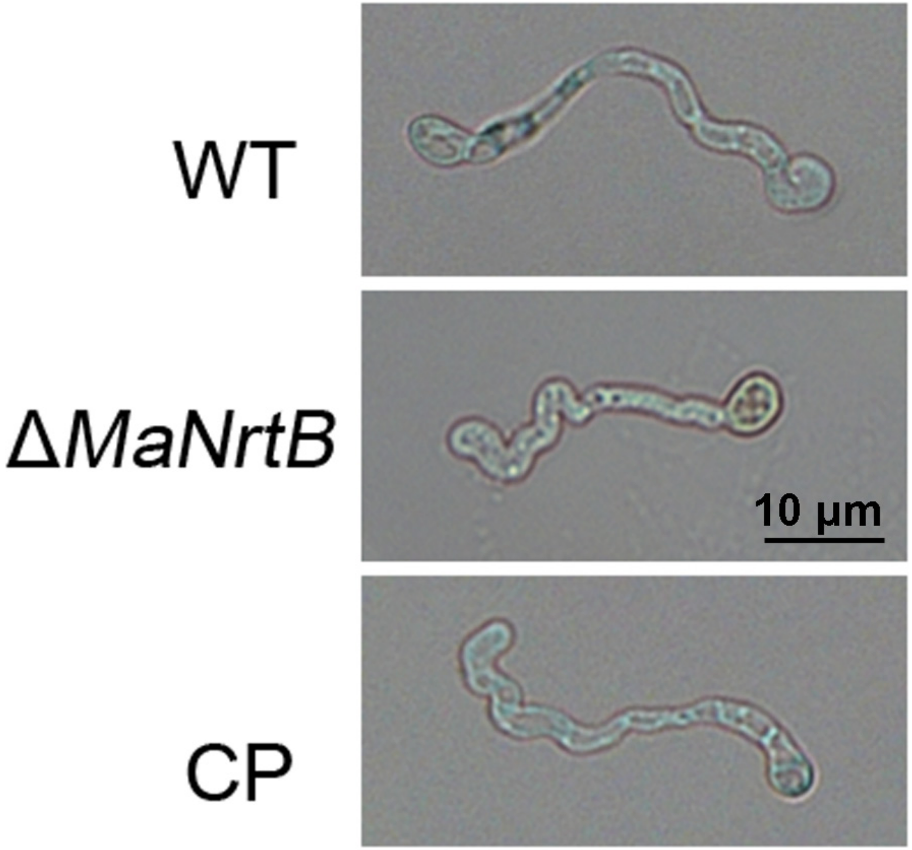

Figure S5 Photomicrographs of appressoria formed by WT, Δ*MaNrtB* and CP. All three strains were able to form normal appressorias, but there were no obvious differences in their morphology.

Table S1 Primers used in this study.

Primers	Sequence (5′→3′)	Amplication size	Remarks
NrtB-LF	gacggccagtgccaagctAACGCCACCAAGAAGGTAT	1272 bp	Used to construct the *MaNrtB*-disruption vector. Lowercase letters represent the adapter sequences added to the primer.
NrtB-LR	cggatccctcgagtctagATTGATTTGAGTTGTTGGCG		
NrtB-RF	gctggccgcccatgggatTTCATATTGCCGTCAACTTG	1215 bp	
NrtB-RR	atgacatgattacgaattGATATTGGCACTGATTGGAA		
NrtB-VF	TCGAGCAGCTTCAACTTATA	1413 bp	Used to screen the *MaNrtB*-disruption transformants
Pt-R	CAGCCAAGCCCAAAAAGTG		
NrtB-VR	ATACGATACGATACATGGTG	1527 bp	
Bar-F	GCTCTACACCCACCTGCT		
EGFP-F	ctagactcgagggatccgCGGGCAGGATCCATGGTGAGCAAGG	726 bp	Used to construct the pk2-eGFP-sur vector
EGFP-R	CGTTAAGTGGATCCTTATCA		
TtrpC-F	GGATCCACTTAACGTTACTG	719 bp	
TtrpC-R	ccttgctcaccatggatccAAGAAGGATTACCTCTAAAC		
NrtB-CP-F	gacggccagtgccaagctGAAGAAAAGTTGATGCACAC	4597 bp	Used to construct the *MaNrtB*-CP vector. Lowercase letters represent the adapter sequences added to the primer.
NrtB-CP-R	ccttgctcaccatggatccCTGCCCGCCGACTTGGCCCT		
NrtB-CP-VF	CGATGTCATAGCCTTATGGT	3372 bp	Used to screen the *MaNrtB*-CP transformants
GFP-VR	CGATGCGGTTCACCAGGGTGT		
NrtB-qF	TTTTCTTCTCTTGGATGGGG	134 bp	Used to analyze the expression of *MaNrtB*

NrtB-qR	GTCAACAATGGAGGAAACGT		by qRT-PCR
gapdh-qF	GACTGCCCGCATTGAGAAG	149 bp	Used to analyze the expression level of *gapdh*
gapdh-qR	AGATGGAGGAGTGGGTGTTG		
Defensin-qF	GCGTCTGTCTCCTCTG	152 bp	Used to analyze the expression level of *Defensin*
Defensin-qR	CCCTTGTAGCCCTTGTT		
Attacin-qF	GTGCTCCTCGTCGTTCTGA	113 bp	Used to analyze the expression level of *Attacin*
Attacin-qR	CCCACGCCTTTCTCTCTGT		
β-actin-qF	CGAAACCTTTAATACCCCAG	102 bp	Used to analyze the expression level of *β-actin*
β-actin-qR	CCATCACCAGAATCCAACAC		
TEF-F	CAGAGAGTAAGTTGCGTGAG	2125 bp	Used to clone the *TEF* gene in *M.acrdum*
TEF-R	GTCAACGGGCATCGGCATT		

Table S2 Data of fungal biomass measurements in different media.

Medium	WT	Δ*MaNrtB*	CP
SPM	0.4152, 0.4620, 0.4440	0.3413, 0.3737, 0.3112	0.3856, 0.4212, 0.4581
CZA	0.0093, 0.0105, 0.0114	0.0042, 0.0051, 0.0043	0.0084, 0.0094, 0.0093
TPM	0.4174, 0.4773, 0.3898	0.2325,0.2872, 0.2172	0.3258, 0.3962, 0.3727
